# Dreams and Dissociation—Commonalities as a Basis for Future Research and Clinical Innovations

**DOI:** 10.3389/fpsyg.2020.00745

**Published:** 2020-04-22

**Authors:** Dalena van Heugten-van der Kloet, Steven Jay Lynn

**Affiliations:** ^1^Department of Clinical Psychological Science, Faculty of Psychology and Neuroscience, Maastricht University, Maastricht, Netherlands; ^2^Psychology Department, Binghamton University (SUNY), Binghamton, NY, United States

**Keywords:** dissociation, dreaming, consciousness, sleep-wake cycle, sociocognitive model

Dissociative symptoms refer to a spectrum of non-ordinary disruptive experiences from “zoning out,” to out-of-body experiences, to outright distortions in the fundamental sense of self, with Dissociative Identity Disorder (DID) as its most debilitating manifestation (Holmes et al., 2005). Dissociative symptoms range from 1 to 3% among general population and from 4 to 14% among psychiatric patients (Sar, 2011). In psychiatric patients, dissociative symptomatology can have a serious impact. Mean impairment scores of patients with dissociative disorders on measures of psychosocial, occupational, and interpersonal functioning are >50% higher than those of patients with other mental disorders (Mueller-Pfeiffer et al., 2012), and dissociative symptomatology is strongly related to self-harm and multiple suicide attempts (Foote et al., 2008). Relative to 17 other mental disorders, patients with dissociative disorders consumed the highest number of outpatient therapy sessions (Mansfield et al., 2010). Importantly, although dissociative symptoms are most salient and persistent in dissociative disorders such as DID, they are considered transdiagnostic phenomena and comorbid with many other conditions (e.g., psychotic illness, anxiety, depression).

No evidence-based treatment consensus exists for dissociative disorders due to lingering controversies. Two perspectives, the trauma model and sociocognitive model, have vied for acceptance and empirical support over decades. The trauma model posits a causal relation between trauma and dissociative symptoms (Dalenberg et al., 2012; Vissia et al., 2016). Accordingly, dissociation is viewed as a coping mechanism triggered by childhood trauma in which distinct personality states, for example, arise to detach from emotionally overwhelming memories (Van der Hart et al., 2006).

In contrast, the sociocognitive model contends that dissociative symptoms are shaped by social learning and cultural expectancies regarding clinical features of dissociation, as portrayed by media and reified by inadvertent therapist cueing. The model assumes that vulnerable patients come to adopt a narrative of being populated by distinct selves to explain mood swings, impulsive actions, and other puzzling behaviors (Lynn et al., 2019). Rapprochement between these models is needed and could be facilitated by fundamental research that clarifies antecedents and correlates of dissociation, including co-occurring sleep problems, that would potentially facilitate treatment consensus and innovation.

## Dissociation and Sleep

Previous studies have secured moderate-to-high correlations of dissociative symptoms with sleep disturbances as well as provided evidence for disturbed sleep playing a causal role in dissociative symptoms (Watson, 2001; Van der Kloet et al., 2012a; Merckelbach et al., 2017; Schimmenti, 2017): Whereas sleep loss induces dissociative symptoms (Van Heugten – Van der Kloet et al., 2015) sleep improvement, in contrast, reduces dissociative symptoms (Van der Kloet et al., 2012a), indicating an association of a labile sleep-wake cycle with both acute and chronic sleep disturbances and dissociative symptoms among healthy and clinical populations.

An important theoretical and research question is whether dissociative symptoms, which range on a continuum of severity, are triggered by disruptions in memory and metacognitive processing that occur during sleep states, with disruptions during REM sleep of particular relevance, that carryover to waking life. When sleep and dream systems become impaired, memory processes during (REM) sleep become dysregulated and engender information overload from internal and external sources that (a) overwhelms cognitive processing, (b) impairs integration of self-relevant information and memories, and (c) induces dissociative symptoms, which are potentially manifested in fragmented (i.e., dissociated), dream-like mentation, illusions, delusions, memory distortions, and, ultimately, a disturbed sense of self (McNamara, 2013). Dreamlike phenomena, which are ordinarily confined to sleep, thus intrude into waking consciousness and are expressed as dissociative symptoms, including depersonalization and derealization, and, in the extreme case, identity fragmentation evident in DID.

## Consciousness and Dreaming

Conscious states may be defined as representations of brain states that arise as a function of shifting dynamics of large-scale neuronal networks (Freeman, 2000; Varela et al., 2001; Bob and Louchakova, 2015). (Libet, 2006) posited that subjective experience is represented in the brain by synchronized activities of large numbers of neurons, referred to as a “cerebral mental field.” This conceptualization affords description of subjective experiences in terms of constantly morphing brain activation patterns that not only generate consciousness via intricate feedback loops, but consciousness, itself, reciprocally affects brain dynamics. Neural systems thus create mental representations of perception, cognitive functioning, memory, and consciousness more broadly (Freeman, 2000; Singer, 2001). Interestingly, stressful experiences can affect the neural mechanisms that enable integration of contents of consciousness, potentially fueling dissociation of conscious awareness and memory (Bob, 2003; Spiegel, 2012) and disrupting sleep.

What is the role of dreams in processes related to dissociation (failure to integrate mental content into conscious awareness), defined conventionally as: “a disruption of and/or discontinuity in the normal, subjective integration of one or more aspects of psychological functioning, including—but not limited to—memory, identity, consciousness, perception, and motor control” [DSM-5; American Psychiatric (American Psychiatric Association., 2014)]. Dissociative states not only occur during wakefulness among healthy individuals and those with mild dissociative symptoms, but they are also manifested during dreams, typically related to shifts in dream scenes and particularly during nightmares and recurrent dreams (Hartmann, 1998; Bob, 2004; Schonhammer, 2005). Among 43 patients diagnosed with dissociative identity disorder (DID), 57% indicated that their “alter personalities” presented as dream characters in their dreams (Barrett, 1994). Dream characters can be viewed as hallucinated projections of the fragmented self; dreaming, in turn, may reflect dissociative states represented during memory processing in REM sleep (Bob, 2004; Stickgold and Walker, 2005).

In contrast with the synchronized activity of large groups of neurons in the “cerebral mental field,” in some states of consciousness, such as dreaming, meditation, divergent thinking, and dissociative states, neural network patterns may function in a more chaotic, unstable, and non-linear fashion (Kahn and Hobson, 1993; Bob, 2003) in which a small perturbation in the system can resonate and induce large changes in the system's behavior (Bob and Louchakova, 2015). For example, flexibility of mental processes facilitates generating patterns that create the subjective experience of coming up with “novel” ideas (Freeman, 2000). During chaotic brain states, activities usually take place in various regions of the brain acting simultaneously but independently. When the strength of the associations and information processing systems among these regions is greatly attenuated or impoverished and mental contents become fragmented and disorganized, dissociated mental states may arise (Bob, 2003). The sudden transitions of dream objects and sceneries experienced in dreams, may reflect dissociation related to rapid shifts in neural patterns related to chaotic or—as they are also called—self-organizing neural activities, mainly stemming from the pontogeniculo-occipital (PGO) systems in the brain (Kahn and Hobson, 1993).

## Lucid Dreaming

A particular type of dreaming may be of special interest in this respect: lucid dreaming. According to Voss and Hobson (2014), insight, control, and dissociation represent the defining criteria for lucid dreaming. Insight refers to metacognitive reflective thought, i.e., the dreamer is aware that she is dreaming, and it is considered the core criterion. Control allows the dreamer to change the dream plot, and dissociation happens when the dreamer experiences the dream as feeling unreal (similar to waking derealization) or sees herself from a distance [similar to waking depersonalization; (Voss et al., 2018)]. This third person perspective can also entail the dream experience itself. Dreamers then experience the dream sequence from the outside, as if the dream were a movie. By this definition, lucid dreaming can be viewed as “a dissociative mental state of consciousness in which the dream self separates from the ongoing flow of mental imagery.” (Voss et al., 2018, p.3). However, in lucid dreams a sense of reality or awareness of dreaming is superimposed on the “unreality” of the dream, whereas in depersonalization/derealization, a sense of unreality is superimposed on the “reality” of mundane waking existence. Thus, in lucid dreams meta-consciousness is preserved to a greater extent than in non-lucid dreams, whereas in depersonalization/derealization, meta-consciousness of the self and the surround is compromised relative to everyday normative experiences. These differences between lucid dreaming and dissociative experiences might explain why the correlation between measures of lucid dreaming and dissociation, while statistically significant, is weaker than the correlation between unusual sleep experiences (e.g., sleep paralysis, hypnagogic hallucinations, nightmares) and dissociation (Van der Kloet et al., 2012a). We suggest that such “dream-like” experiences infiltrate waking consciousness to create an experience of unreality that is expressed as dissociative experiences and symptoms.

Can dissociation be experienced as beneficial? Dissociation is usually transient during waking and associated with daydreaming and fantasy proneness in healthy adults (Van der Kloet et al., 2012b), at the mild end of the dissociation continuum. In the context of psychiatric diagnoses, some theorists have described dissociation as a protective mechanism to cope with emotional pain in posttraumatic stress disorder via downregulation of the limbic system, thereby suppressing unconscious affect (Lanius et al., 2010) and enabling self-conscious emotions via activation of the ventral prefrontal cortex [VPFC; (Damasio, 1988)]. In psychosis, dissociation is often undesirably associated with positive symptoms. However, Dalle Luche (2002) advanced a nuanced view by proposing that dissociative thought is more fleeting in the early stages of psychosis, whereas the loss of a sense of self is more prominent in the later stages of illness. Viewed in this light, dissociative cognition in lucid dreaming mirrors the type of dissociation experienced in the early stages of psychosis. Although attempts to control dream content can disturb sleep, an increase of lucid dreaming and accompanying dissociative thought may also be desirable as the heightened insight/meta-consciousness may be associated with a weakening of psychosis-like experiences. In general, lucidity in dreaming has been linked with positive rather than negative emotions. In normal REM dreaming, due to attenuation of the VPFC, unconscious emotions take the stage. In lucid dreaming, with the VPFC switched on again, self-conscious emotions take the lead and unconscious emotions are down-regulated. This process engenders an overall reduction of emotionality compared with regular dreams (Voss et al., 2018). Indeed, dissociative thought seems to down-regulate negative emotion both in dreaming as during wake (LaBerge and Rheingold, 1991; Voss et al., 2013), with parallels in lucid dreaming and psychiatric illness [but see (Mota et al., 2016)].

## Fodder for Future

Our discussion implies that it is possible to enhance insight and meta-consciousness via lucid dreaming in patients suffering from psychiatric disorders such as in dissociation and psychotic illness, in order to reduce negative emotions. Training the frontal lobe explicitly to create insight in the delusional feature of a dream may provide a foundation of enhancing reflective thought during the daytime as well. Indeed, researchers have piloted lucid dreaming as a clinical treatment in various groups with mixed results (Spoormaker and Van den Bout, 2006; Lancee et al., 2010). However, we suggest that therapists make explicit the purpose of enhancing meta-consciousness across the entire sleep-wake continuum to enhance generalizability of outcomes across the sleep/wakefulness spectrum and continuum of severity of dissociative symptoms. Notably, researchers have successfully treated patients with dissociative identity disorder with transdiagnostic interventions geared to improve sleep, enhance meta-consciousness, and emotion regulation, and decrease fragmentary, hyperassociative thinking that marks both dissociative conditions and dream consciousness (Mohajerin et al., 2019).

Treatment costs of patients with dissociative psychopathology are very high, while psychological interventions are generally not evidence-based and innovative treatments stagnate due to lingering controversies across theoretical camps. This state of affairs also impacts innovation in treating psychiatric conditions (e.g., PTSD, borderline personality disorder, schizophrenia spectrum disorders) with high comorbidity with dissociative conditions [see (Lynn et al., 2019)]. Moreover, dissociative comorbidity is a severity marker signaling poor prognosis.

As ineffective and non-optimal treatments impose considerable burdens on patients and society, novel research programs focusing on the relations among dissociation, the sense of self, and sleep and dreaming are a priority. Studying both the chaotic and the deterministic brain state during sleep and wakefulness may provide insight into important functions of perception, memory, and cognition and what happens when they become dissociated. In doing so, the study of dissociation may provide important clues regarding the nature of human consciousness itself. Importantly, this effort will inform clinicians and researchers alike and serve as an impetus for new treatment studies, including research evaluating interventions targeting dissociative psychopathology via enhancing sleep and metacognitive processing.

## Author Contributions

DH wrote the original version of the manuscript, which was edited by SL.

## Conflict of Interest

The authors declare that the research was conducted in the absence of any commercial or financial relationships that could be construed as a potential conflict of interest.
